# The Role of Mirk Kinase in Sarcomas

**DOI:** 10.1155/2011/260757

**Published:** 2011-04-13

**Authors:** Eileen Friedman

**Affiliations:** Department of Pathology, Upstate Medical University, 750 East Adams Street, 2305 Weiskotten Hall, Syracuse, NY 13210, USA

## Abstract

Targeting the tyrosine kinase KIT in gastrointestinal stromal tumors has led to improved treatment. Other kinases might serve as therapeutic targets in the more common forms of sarcoma. The kinase Mirk/dyrk1B is highly expressed in the vast majority of osteosarcomas and rhabdomyosarcomas and mediates their growth, as depletion of Mirk led to tumor cell apoptosis. Mirk is known to increase the expression of a series of antioxidant genes, which scavenge reactive oxygen species (ROS) within various tumor cells, mediating their survival. As a result, depleting Mirk led to increased levels of damaging ROS. Tumor cells depleted of Mirk were also sensitized to low levels of chemotherapeutic drugs that increase ROS levels. In contrast, Mirk expression is quite low in most normal cells, and Mirk depletion or embryonic knockout of Mirk did not detectably affect cell survival. Thus targeting Mirk for intervention in sarcomas might spare most normal tissues.

## 1. Introduction

Targeting of cellular kinases has proved efficacious for the treatment of various cancers. Kinases are a good target for therapy because they are readily inhibited by small, cell permeable molecules that block their ATP-binding site and because they act catalytically, and so they are in relatively low abundance compared to structural elements within a cell. In gastrointestinal stromal tumors (GISTs), the use of inhibitors of the stem cell factor receptor kinase, KIT has dramatically impacted treatment (reviewed in [1, 2]. The tyrosine kinase KIT is expressed in more than 95% of GISTs, with many exhibiting mutations that increase kinase activity. The Kit inhibitors imatinib and sunitinib have induced stable disease or partial responses in many patients, increasing their length of survival. While GISTs represent only about 5% of all sarcomas, the efficacy of treatment with KIT kinase small-molecule inhibitors suggests that other kinases may represent targets in more prevalent sarcomas.

Mirk/Dyrk1B is a member of the Minibrain/dyrk family of serine-threonine kinases [3–5]. Mirk is expressed at very low levels in most normal tissues [6]. However, Mirk is highly expressed in normal skeletal muscle and in C2C12 myoblasts where it mediates differentiation and survival. Mirk aids in the differentiation of skeletal muscle [7] and maintains the survival of differentiating myoblasts [8]. Mirk is not an essential gene because embryonic knockout of Mirk/dyrk1B caused no evident phenotype in mice [9]. Likewise, normal diploid fibroblasts exhibited no alteration in survival after 20-fold depletion of Mirk [10], suggesting that targeting Mirk for intervention might induce a selective killing of tumor cells.

## 2. Mirk in Osteosarcomas

Osteosarcoma is the most common malignant bone tumor and is highly metastatic. After chemotherapy, the tumor recurs in about one-third of patients and the life expectancy after recurrence is less than one year [11, 12]. Cytoplasmic kinases and growth factor receptor kinases have been implicated in sarcoma survival including mTOR [13, 14], PDGFR-A [15], and the IGFR1 [16, 17]. Recently an RNA interference screen of the osteosarcoma cell line KHOS was performed using a lentiviral short hairpin RNA library targeting 673 human kinase genes [18]. The Mirk gene was found by this screen to mediate sarcoma cell proliferation and apoptosis, while a Mirk cDNA rescue assay confirmed that the identification of Mirk was not due to off-target effects. Mirk knockdown by shRNA or by synthetic RNAi duplexes induced apoptosis in each of 3 osteosarcoma lines tested as well as an osteosarcoma in primary culture. No effect was seen on a benign osteoblast cell line. Mirk protein was widely expressed in this cancer, being found in each of 58 osteosarcomas in a tissue microarray. Most significantly, Kaplan-Meier analysis showed that patients whose tumors expressed the highest amount of Mirk protein had significantly worse prognosis than those with low Mirk expression. For this analysis, patients were stratified into two groups, those alive up to 60 months after followup and those deceased. The nonsurvivors had more Mirk in their osteosarcomas, with *P* = .0012. This report [18] indicated that the kinase Mirk was essential for the growth and survival of osteosarcoma cells and that high Mirk protein levels in the cancer were a biomarker for tumor progression.

## 3. Mirk in Rhabdomyosarcomas

Rhabdomyosarcoma is the most common soft tissue sarcoma in children and is difficult to treat if the primary tumor is nonresectable or if the disease presents with metastases [19, 20]. There are two major histological types, embryonal and alveolar. Alveolar histology is associated with a significantly worse prognosis with a five-year survival rate of less than 30%. The precise etiology of rhabdomyosarcoma is unknown, but it has been suggested to arise in “satellite” cells, the committed skeletal muscle precursor cells [19]. Mirk/Dyrk1B was expressed to some extent in each of 16 clinical cases of human rhabdomyosarcoma examined [21] and in myoblast satellite cells [7]. Furthermore, Mirk was found to be an active kinase in each of 3 rhabdomyosarcoma cell lines tested [21]. In addition, Mirk depletion by synthetic RNAi duplexes induced apoptosis in each of two rhabdomyosarcoma cell lines assayed as shown by increase in both the apoptotic marker Annexin V and DNA breaks revealed by TUNEL staining. Increased apoptosis led to a 3-4-fold decrease in clonogenicity. Thus depleting Mirk led to death of the most aggressive rhabdomyosarcoma cells.

## 4. Mirk in Skeletal Muscle Myoblasts

Some insight into the possible role of Mirk in rhabdomyosarcoma can be derived from studies of Mirk in skeletal muscle myoblasts. Mirk was expressed in skeletal muscle satellite cells in primary culture and was upregulated about 10-fold when the satellite cells were induced to differentiate, while knockdown of endogenous Mirk by RNA interference blocked myoblast differentiation [7]. Mirk is activated by the stress-activated MAP kinase kinase MKK3 [22]. These results together imply a role for Mirk in the response to cellular injury. Skeletal muscle is regenerated after injury by activation of quiescent satellite cells that enter the cell cycle and then differentiate and fuse with uninjured muscle fibers to repair the damage. Mirk may play some role in muscle regeneration because Mirk is a stress-activated kinase that modulates the activation of the myogenic regulatory factors MEF2 and myogenin, which subsequently mediate myoblast differentiation [8]. Mirk is less likely to play a significant role in embryonic muscle development because a Mirk/Dyrk1B knockout mouse survived to 18 days after conception during which time skeletal muscles were developed [9]. Thus Mirk/Dyrk1B may be a survival factor in skeletal myoblasts undergoing repair.

## 5. Inactivation of ROS May Be the Mirk Survival Function in Sarcomas

The Mirk kinase gene has been localized to the 19q13 amplicon [6] and is amplified in a subset of pancreatic cancers and ovarian cancers, and less frequently in colon cancers [23–25]. Mirk mediates survival of these cancers at least in part by reducing reactive oxygen species (ROS). ROS are oxygen-containing chemical species with reactive chemical properties, such as hydroxyl radicals, which contain an unpaired electron and the free radical superoxide. Cancer cells often exhibit higher levels of ROS than normal cells because of increased metabolism and oncogenic stimulation, and so they are under increased oxidative stress. Genes which detoxify superoxide (superoxide dismutases 2 and 3) and which prevent the generation of hydroxyl radical (ferroxidase/ceruloplasmin) were found to be upregulated in SU86.86 pancreatic cancer cells [26] and in each of four ovarian cancer cell lines [27] through Mirk. These genes work together to reduce ROS. Superoxide dismutases detoxify superoxide resulting in hydrogen peroxide, which in turn can be metabolized either to water or to hydroxyl radical through the Fenton reaction if Fe++ is available. Conversion to hydroxyl radical is blocked by ferroxidase that converts Fe++ to Fe+++. Mirk is a coactivator for several transcription factors and increases the expression of these antioxidant genes [26]. Thus these Mirk-upregulated genes working together increase antioxidant potential while minimizing hydroxyl production.

## 6. ROS in Skeletal Muscle

ROS are toxic to cells, decreasing their viability; so ROS levels and cell viability fell following depletion of Mirk from C2C12 myoblasts and from cancer cells. Using immunofluorescence techniques, we have found that Mirk is localized in fast twitch skeletal muscles (Mercer and Friedman, manuscript in preparation). Such muscle endogenously produces ROS in response to repeated contractions. Hydrogen peroxide is produced in contracting muscle, breaking down to ROS species, which can have diverse effects on myoblasts, such as inducing mitochondrial fragmentation [28]. ROS generation within single intact muscle fibers was cytosolic, with a role for NADPH oxidase-derived ROS during contractile activity [29]. Depletion of Mirk from C2C12 myoblasts also led to an increase in ROS (Deng and Friedman, manuscript in preparation), consistent with ROS control being a major role of Mirk in muscle development and function. This protective ROS-decreasing role is likely to have provided a selective pressure to maintain elevated Mirk levels in skeletal muscle and to further upregulate Mirk expression in sarcoma cells. Thus we hypothesize that Mirk mediates sarcoma cell survival through an increase of its original function in skeletal muscle cells, depletion of ROS.

## 7. Mirk Depletion/Inactivation Potentiates Certain Chemotherapeutic Drugs

The chemotherapeutic drug cisplatin is one of many known to increase intracellular levels of toxic reactive oxygen species. Thus, an increase in cisplatin toxicity selectively in cancer cells could result from further increasing the cisplatin-elevated ROS levels by targeting antioxidant genes upregulated in cancers such as those mediated by the kinase Mirk/dyrk1B. This possibility was tested, and depletion of Mirk increased cellular ROS levels in each of 4 ovarian cancer cell lines. Mirk depletion averaged only about 4-fold, yet combined with cisplatin treatment enabled low levels of drug to increase ROS to toxic levels in both SKOV3 and TOV21G ovarian cancer cells [27]. Lowering ROS levels by treatment with N-acetyl cysteine limited cisplatin toxicity, resulting in higher cell numbers and decreased cleavage of the apoptotic proteins PARP and caspase 3. Targeting Mirk in sarcomas could increase their response to lower levels of chemotherapeutic drugs, potentially reducing side effects, which often limit therapeutic options in these cancers.

## 8. Hedgehog Signaling in Sarcomas

Mirk/dyrk1B and Dyrk1A are about 94% identical/homologous within their conserved kinase domains, but unlike within their unique N and C termini. The kinase domain similarity has led many to suspect some common functions between Dyrk1A and Mirk/dyrk1B. Dyrk1A, as one of the Down Syndrome conserved genes, has been intensively investigated. The essential embryonic signaling pathway, Hedgehog, has been implicated in many cancers such as pancreas, lung, and prostate, and Gli1 is a target of this pathway. In initial studies Dyrk1A enhanced Gli1-dependent gene transcription and acted synergistically with Sonic hedgehog to induce transcription [30]. However, the involvement of Mirk in Hedgehog signaling is complex. Mirk is activated by oncogenic K-ras and H-ras [10] and is an active kinase in pancreatic cancers [31], which exhibit a very high rate of K-ras mutation, almost 100% in advanced lesions. Mutant K-ras signaling through Mirk/dyrk1B blocked autocrine Hedgehog signaling to Gli1 within pancreatic cancer cells, only allowing Hedgehog signaling to Gli1 in stromal cells, which do not have mutant K-ras [32]. This is important clinically because most drugs do not reach pancreatic cancer cells because of their dense stroma [33], so the paracrine hedgehog signaling in stromal cells can be targeted [34] to enhance conventional chemotherapy. Activation of the hedgehog pathway confers a poor prognosis in embryonal and fusion gene-negative alveolar rhabdomyosarcoma [35], and the transcription factor Gl1 is a central mediator of EWS/FLI1 signaling in Ewing sarcoma tumors [36]. Since Mirk was found to be an active kinase in each of 3 rhabdomyosarcoma cell lines tested [21], it may also alter Hedgehog signaling to a paracrine mode and thus mediate control of the stromal microenvironment of these tumors. The WD40 repeat protein Han11 can inhibit Dyrk1A-dependent transcription of Gli1 when Han11 also binds the cytoskeletal regulator mDia [37]. Mirk/dyrk1B is found in a 670 kDa complex with unknown proteins [38]. One may be Han11, which binds to Dyrk1A, Dyrk1B/Mirk, the related kinase HIPK2, and the mitogen-activated protein kinase kinase kinase1 (MEKK1) [39]. When downregulated, or conversely when overexpressed, Han11 alters the threshold and amplitude of kinase signaling by HIPK2 and MEKK1, demonstrating a scaffolding function for Han11 in controlling these kinases in a multiprotein complex.

## 9. Additional Mirk/Dyrk1B Substrates

Several other intriguing Dyrk1A substrates have been identified (CREB, STAT3, and NFAT) [40–43], and have yet to be examined as potential Mirk substrates in sarcomas. The STAT3 signaling pathway is constitutively activated in each of three rhabdomyosarcoma cell lines tested, and two small-molecule compounds inhibited both STAT3 activity and cell proliferation and viability [44]. Mirk and Dyrk1A are coactivators of FOXO1a-dependent glucose-6-phosphatase gene expression [45], and Dyrk1A phosphorylates this transcription factor [46]. Mirk also slightly increased the activity of FOXO3a on a promoter-reporter construct of the CDK inhibitor p27 [47]. The functional relevance of these interactions is unclear. However, Mirk stabilizes p27 by phosphorylation [48], and so it might augment this activity by increasing p27 expression. Increased p27 levels mediate a G0 arrest where damaged cells can repair [49]. A small-molecule Mirk kinase inhibitor would be very useful in confirming the role of putative Mirk substrates in sarcomas.
